# Supplementary material for: Impairment of visual and neurologic functions associated with agrochemical use
Source: PLoS One. 2023 Aug 23;18(8):e0290263. doi: 10.1371/journal.pone.0290263 (PMC10446217; doi:10.1371/journal.pone.0290263)
Supplement: Attachment — Submitted filename: paper comparing the effect of a wide range of agrochemicals on visual functions.docx [file pone.0290263.s002.docx]

This a good paper comparing the effect of a wide range of agrochemicals on the low visual functions (color vision and contrast sensitivity) and high visual functions (global form perception and motion coherence threshold) among agricultural workers who were frequently exposed to these substances and the age-matched controls who were less frequently exposed.

Mechanism of exposure was either by direct contact with ocular surface of by inhalation and subsequent neurotoxicity when the substance gets to the brain.

The degree of exposure was measured using the modified Q16 questionnaire to assess and compare neurotoxicity score between the exposed and non-exposed groups. The tear film substance P level and visual tests were also done. However, it was not definitively determined, which chemical is the direct cause of the reported symptoms and the visual changes. The research reported an association between exposure to occupational levels of agrochemicals, including pesticides, and alteration in high and low visual functions.

The topic may be modified to better convey the methodology, it may be rendered as “Impairment in high and low visual functions associated with agrochemical exposure: a controlled study “.

There is need for references to be provided to support the assertion that several studies have been done to recognize the deleterious effect of pesticides on brain function in the introduction.

Reference(s) need(s) to be provided to give more information on the Modified-16Q questionnaire and how it is scored in assessing neurotoxicity of pesticides. No search material matches the reference provided.

To aid reproducibility of this research, the distance of the place of residence of controls stated as “sufficiently away from agricultural farms” should be specified. So also, the estimated maximum number of times of exposure of the controls to the agrochemicals should be stated.
