# Supplementary material for: Impairment of visual and neurologic functions associated with agrochemical use
Source: PLoS One. 2023 Aug 23;18(8):e0290263. doi: 10.1371/journal.pone.0290263 (PMC10446217; doi:10.1371/journal.pone.0290263)
Supplement: Attachment — Submitted filename: Response to Reviewers.docx [file pone.0290263.s003.docx]

Dear, Dr Samuel Adelani Babarinde

Thank you for handling the review of our manuscript. We thank you and the reviewers for their constructive comments. In the interest of providing a clearer submission, changes have been made to our manuscript in line with their suggestions. These changes are detailed point by point below. Hopefully with these changes a decision can be made regarding the suitability of our manuscript for publication in PloSONE.

**Reviewer #1 comments:**

1. Title- The article is not only about the visual function of the participants but it is also assessing the neurotoxic symptoms so this should be captured in the topic to be something like ‘Impairment of visual and neurologic functions associated with agrochemical use.’

**Answer:** We agree that the title requires change to better reflect the aims and findings of the study. Both reviewers suggested appropriate titles, and in discussion with the authors we agree that the title suggested by reviewer 1, is a better description of the study. The title now has been changed.

2. The presence of Substance P in the tear film actually shows that there is an ocular surface inflammation going on, but this only could not specifically be a measure of ocular surface damage because the amount of it’s presence in individuals could be affected by other factors. A better measure of the ocular surface damage should be done using Slip lamp biomicroscope of the cornea in which epithelial defect can be identified or any other defect on any layer of the cornea OR you do an Anterior Segment Optical coherence tomography or any other imaging modality to assess the ocular surface.

**Answer:** The subtitle in lines 157-158 has been changed to “Measures of neurotoxicity exposure and levels of substance P in tears”.

3. This study assessing the effect of organic solvents on ocular surface of workers should be a prospective cohort study and not a cross-sectional study. Although controls were used but all these tests (visual and neurologic) were not done for the participants before stating the work on the agricultural farm. So how are you sure your findings were developed due to the exposure of the agrochemicals? Some might have had a mild premorbid ocular condition that does not warrant any complaints before so using verbal complaints are not enough. Subjective self-assessment of the ocular status is not enough because it can be different in different individuals.

**Answer:** Note that in the present study, there was no “study period” and the participants were not measured at multiple time points. In our study, participants were measured only at once, and comparisons were made between the exposed and non-exposed groups at only one point. Therefore, it was not a prospective cohort study, but rather a cross-sectional case-control study. We raise the limitations of this design in the general discussion to alert readers to the possibility that symptoms and visual dysfunction may have existed prior to pesticide exposure (lines 485-490).

4. Are the two groups sex and age matched? Because some of the tests, especially the contract sensitivity test, varies with age. So, if there is marked age difference then the comparison will be faulty.

**Answer**: The average age of the exposed group was 35.2 years (SD±9.2 years), and 82.0% were women (line 289-290). In the control group, the average age was 30.69 years (SD±8.3 years), and 82.05% were women (line 305-306). According to the data, the two groups are matched.

5. The findings on the visual acuity was not reported at all in this article…. Is it similar in the two groups? Because poor visual acuity will definitely after colour vision and contrast sensitivity. And Visual acuity is the main measure of visual function, all other tests are secondary. Was it affected at all in this study?

**Answer**: In the study, all visual tests for both cases and controls were performed with optical correction. The worst best-corrected visual acuity accepted in all participants was 0.1 log MAR. This point is clarified in line 187-189.

6. Who did the direct ophthalmoscopy and who interpreted the images from the digital retinograph system? These assessments need to be reviewed by an ophthalmologist before you can say that the findings are essentially normal. Because any of them might have Retinal dystrophy that is not yet symptomatic, and such is better identified by an ophthalmologist.

**Answer:**  Direct ophthalmoscopy and the images were evaluated by two optometrists’ researchers with PhD in Optometry and Master's in Vision Science, both under the guidance of a retinal ophthalmologist.

7. Did the author provide spectacles for all those who have refractive errors free of charge? Or are they all on insurance that can ensure they get their spectacles without paying out of pocket because ‘cost’ can be barrier to uptake of spectacle use.

**Answer:** All participants have health insurance that cover their spectacles. By law, companies are required to affiliate their workers with the health security system, which includes health insurance coverage.

8. The authors did not mention the procedure and who took the biological samples (Blood and tears) Was necessary precaution taken while taking the samples to ensure samples were not contaminated because these can affect the results obtained.

**Answer:** All samples were taken by a certified professional bacteriologist (line 171) who is trained and qualified to perform this type of sampling.

9. How was the modified Q16 neurotoxic symptoms questionnaire administered? Is it self-administered or interview administered? This is important to ensure that all the participants had a good understanding of the questions asked because the participants have different educational levels.

**Answer:** The Q16 neurotoxic symptoms questionnaire was administered through an interview process, with the interviewer recording the participant's response, and providing an opportunity for the participant to ask questions or clarify any doubts. It was not self-administered (as clarified in lines 164-167).

10. The authors did not mention the percentages of the workers that usually uses or don’t use their protective clothing’s while using these chemicals. This is very important because it directly determines the percentages of the workers directly exposed to these chemicals.

**Answer:** As mentioned in lines 130-133, although the use of protective equipment is mandatory by law, all workers reported not always using them. Unfortunately, a checklist was not conducted to confirm the use of protective equipment during each worker's work shift, and no percentages of usage of these elements were mentioned. This issue is now mentioned in the revised manuscript (see lines 130-133).

**Review #2 comments:**

1. The topic may be modified to better convey the methodology; it may be rendered as “Impairment in high and low visual functions associated with agrochemical exposure: a controlled study “.

**Answer:** The title has been changed to “Impairment of visual and neurologic functions associated with agrochemical use” as suggested by reviewer 1 as we feel that this more appropriately describes the present study.

2. There is need for references to be provided to support the assertion that several studies have been done to recognize the deleterious effect of pesticides on brain function in the introduction.

**Answer:** Additional references were added in line 72, and posteriorly, we mention more studies in line 83 and line 85.

3. Reference(s) need(s) to be provided to give more information on the Modified-16Q questionnaire and how it is scored in assessing neurotoxicity of pesticides. No search material matches the reference provided.

**Answer:** The links to the three articles are attached here and were added as references 14-16 in line 103 and line 168.

*Reference 14:* Jiménez-Barbosa IA, Boon MY, Khuu SK. Exposure to organic solvents used in dry cleaning reduces low and high level visual function. PLoS One. 2015;10: 1–23. doi:10.1371/journal.pone.0121422.

Available <https://journals.plos.org/plosone/article?id=10.1371/journal.pone.0121422>

*Reference 15:* Jiménez Barbosa ÍA, Khuu S y Ying Boon M. Modificación del cuestionario de síntomas neurotóxicos (Q16). Cienc Tecnol Salud Vis Ocul. 2011;(1): 19-37. <https://ciencia.lasalle.edu.co/cgi/viewcontent.cgi?article=1029&context=svo>

*Reference 16:* Jimenez- Barbosa Ingrid Astrid. Visual Function in Dry Cleaners. The University of New South Wales. 2014. <https://doi.org/10.26190/unsworks/16756> <http://hdl.handle.net/1959.4/53436>.

Available: <http://www.unsworks.unsw.edu.au/primo_library/libweb/action/dlDisplay.do?vid=UNSWORKS&docId=unsworks_12131>

4. To aid the reproducibility of this research, the distance of the place of residence of controls stated as “sufficiently away from agricultural farms” should be specified. So also, the estimated maximum number of times of exposure of the controls to the agrochemicals should be stated.

**Answer:** The controls resided at an approximate distance of 15 km from the farm where the workers were located (line 114-115). The controls were individuals who lived and worked in the city and had no direct contact with pesticides which the level of neurotoxicity was less than 32 in the modified Q16 questionnaire (i.e., less than low level) (line 111-112).
